# Characterising residual malaria transmission in forested areas with low coverage of core vector control in central Viet Nam

**DOI:** 10.1186/s13071-019-3695-1

**Published:** 2019-09-18

**Authors:** Hannah Margaret Edwards, Vu Duc Chinh, Bui Le Duy, Pham Vinh Thanh, Ngo Duc Thang, Dao Minh Trang, Irwin Chavez, Jeffrey Hii

**Affiliations:** 1 Malaria Consortium Asia, Room No. 805, Faculty of Tropical Medicine, Mahidol University, 420/6 Rajavidhi Road, Bangkok, 10400 Thailand; 2 Department of Infectious Disease Epidemiology, Imperial College London, London, UK; 3 National Institute of Malariology, Parasitology and Entomology (NIMPE), 34 Trung Văn, Nam Từ Liêm, Hanoi, Viet Nam; 4 Department of Tropical Hygiene, Faculty of Tropical Medicine, Mahidol University, 420/6 Rajavidhi Road, Bangkok, 10400 Thailand

**Keywords:** Residual malaria transmission, Mobile migrant population, Viet Nam, Forest malaria, Slash and burn farming

## Abstract

**Background:**

Despite great success in significantly reducing the malaria burden in Viet Nam over recent years, the ongoing presence of malaria vectors and *Plasmodium* infection in remote forest areas and among marginalised groups presents a challenge to reaching elimination and a threat to re-emergence of transmission. Often transmission persists in a population despite high reported coverage of long-lasting insecticidal nets (LLINs), the mainstay control method for malaria. To investigate what factors may contribute to this, a mixed-methods study was conducted in Son Thai commune, a community in south-central Viet Nam that has ongoing malaria cases despite universal LLIN coverage. A cross-sectional behavioural and net-coverage survey was conducted along with observations of net use and entomological collections in the village, farm huts and forest sites used by members of the community.

**Results:**

Most community members owned a farm hut plot and 71.9% of adults aged 18+ years sometimes slept overnight in the farm hut, while one-third slept overnight in the forest. Ownership and use of nets in the village households was high but in the farm huts and forest was much lower; only 44.4% reported regularly using a bednet in the farm and 12.1% in the forest. No primary anopheline species were captured in the village, but *Anopheles dirus* (*s.l.*) (*n* = 271) and *An. maculatus* (*s.l.*) (*n* = 14) were captured as far as 4.5 km away in farm huts and forest. A high proportion of biting was conducted in the early evening before people were under nets. Entomological inoculation rates (EIR) of *An. dirus* (*s.l.*) were 17.8 and 25.3 infectious bites per person per year in the outdoor farm hut sites and forest, respectively, for *Plasmodium falciparum* and 25.3 in the forest sites for *P. vivax*.

**Conclusions:**

Despite high net coverage in the village, gaps in coverage and access appear in the farm huts and forest where risk of anopheline biting and parasite transmission is much greater. Since subsistence farming and forest activities are integral to these communities, new personal protection methods need to be explored for use in these areas that can ideally engage with the community, be durable, portable and require minimal behavioural change.

## Background

Malaria in the Greater Mekong Subregion (GMS) has significantly reduced in recent years and Viet Nam has been one of the most successful countries in the region in contributing to this decline. Recent figures from the World Health Organization’s World Malaria report 2017 showed Viet Nam had more than halved the number of malaria cases in 2016 from 2015, from 12,560 to 6000 [1]. The focus is now on understanding and tackling the remaining pockets of malaria transmission which are particularly focused in forested locations where hard-to-reach population groups practice subsistence farming and forest activities.

Malaria vector control in the GMS relies almost exclusively on long-lasting insecticidal nets (LLINs) which reduce malaria parasite transmission mainly by killing or blocking mosquitoes that attempt to feed upon humans sleeping under them. Despite political and donor pressure to distribute LLINs free of charge in all malaria-endemic countries, the Viet Nam National Malaria Control Programme (NMCP) has continued with its re-treatment programme of conventional and insecticide-treated nets (ITNs). This programme has been highly successful in instigating community engagement and social mobilization, as well as providing a platform to expand the reach of information, education and communication (IEC) [2]. This has contributed to the decline in malaria transmission in Viet Nam and to the current transmission landscape where high-risk areas and communities are mostly limited to forested areas dominated by the most common vectors, *Anopheles dirus* and *An. maculatus* [3–5]. These vectors exhibit outdoor and early biting behaviours that overlap with human outdoor early evening activities [6, 7]. As described by Bannister-Tyrell et al. [8] in their study in central Viet Nam, human outdoor activities “may favour exposure to biting vectors that cannot be prevented by sleeping under LLINs…[and]…some risk factors relating to evening outdoor exposure may have been missed in previous studies”.

A vector survey conducted in 2015 in Son Thai commune of Khanh Vinh District, south-central Viet Nam looked beyond the village setting to investigate mosquito biting behaviours in farm huts frequented by the subsistence farming community [9]. Very few *An. dirus* and *An. maculatus* were collected in the village by human landing catch (HLC) but significantly more were captured in farm huts, particularly by outdoor HLC (OHLC) where biting rates were 4.08 bites per person per night (bpn) for *An. dirus* (*s.l.*), 0.17 bpn for *An. maculatus* (*s.l.*) and 0.04 bpn for *An. minimus* (*s.l.*). Concurrent indoor biting rates in farm huts were 0.27 bpn for *An. dirus* (*s.l.*), and zero for *An. maculatus* (*s.l.*) and *An. minimus* (*s.l.*). Since sufficient ITN ownership (the proportion of households with at least one net per two people) was reported to be over 90% in this community but malaria incident cases were still occurring, the remaining transmission could be termed ‘residual malaria transmission’ (RMT), defined as the transmission that remains once universal (> 80%) coverage of LLINs and/or maximal coverage of indoor residual spraying (IRS) has been achieved using insecticides to which the local vectors are susceptible [10]. Coverage is defined as ownership and use of nets based on three basic survey indicators: (i) the proportion of households with at least one ITN/LLIN; (ii) the proportion of population with access to an ITN/LLIN; and (iii) the proportion of the population that slept under an ITN/LLIN the previous night [11]. One problem with the definitions of coverage and RMT is that they focus on village households, yet here and in many high-risk and hard-to-reach communities across the GMS, high mobility and farming practices mean that individuals are at risk not only in their villages, but in other ecological sites such as the farm, forest rest sites and forested waypoints where the key vectors persist [12]. This may help explain the limit of ITNs and IRS among these communities.

We sought to extend this previous vector survey in Son Thai commune using multiple approaches to look at how vector and human behaviours interact to contribute to RMT in an area otherwise poised for malaria elimination. Entomological, epidemiological and observational methods were applied across three ecological sites frequented by individuals in the community, the village, farm huts and forest waypoints, to understand the determinants of RMT first and eventually propose what could be done to aid local elimination of malaria.

## Methods

### Study area

Sites were selected if they met the following inclusion criteria: community has annual malaria cases despite apparent universal ownership of ITNs reported by NMCP/local distribution figures (note that we used ITNs in addition to LLINs since this fits with the Viet Nam NMCP policy of annual net retreatment); communities that practice seasonal subsistence farming/slash and burn agriculture beyond the villages and that travel into the forest; accessible to the survey teams.

**Fig. 1:**
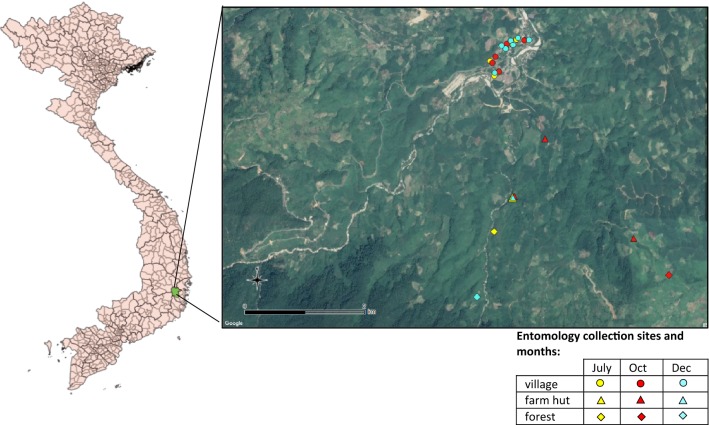
Map of study sites. Left: Map of Vietnam with Khanh Vinh District in green. Right: Map showing location of human landing catch sites in July, October and December, across the three study ecologies

The study took place in the commune of Son Thai (12.2015°N, 108.7482°E) situated in Khanh Vinh District, Khanh Hoa Province, south-central Viet Nam (Fig. 1). Son Thai consists of two closely situated (almost merged) villages called Bo Lang and Giang Bien. Population size in 2016 was 2015 and included mostly individuals from the Coho (Trin) ethnic group (followed by Ra-glai ethnic group). Malaria incidence was reported to be 25.8 and 28.3 per 1000 population in 2015 and 2016, respectively, from local NMCP and health centre records (Khanh Hoa Provincial Health Office, personal communication), and ITN/LLIN coverage was reported to be over 90% [9]. Khanh Hoa Province is mountainous and over half the province is covered by forest while 16.7% is agricultural land (http://www.khanhhoa.gov.vn/). Average monthly temperatures are between 23 °C (December and January) and 27 °C (April and August), and yearly precipitation is between 1400 and 2800 mm, with most rain falling between September and December and the driest months between January and April [13]. *Anopheles dirus* (*s.l.*) and *An. minimus* (*s.l.*) are the primary vector species in the area [9]. Malaria transmission is perennial with two peaks, one in May-June and the other in October-November [14] with increased abundance of *An. dirus* (*s.s.*) and *An. minimus* (*s.s.*) during the rainy months of September to November [9].

### Cross-sectional behavioural and net coverage survey

A cross-sectional household survey was conducted in August 2016 as an add on to community prevalence testing by the NMCP. All households in the commune (*n* = 400) were approached for participation and consent was obtained from the head of the household. Two household members aged > 6 months were randomly selected from a list of householders using a random number generator for prevalence testing. This method is used as standard by the NMCP during all of its community prevalence surveys. Following prevalence testing (data not shown) individuals were questioned regarding household net ownership, as well as personal net use, farm and forest-going habits. Heads of households answered questions on behalf of children and household net ownership was corroborated by the head of the household to ensure accurate household indicator data.

### Data analysis

Data were analysed using Stata v.14 [15] to give proportions and confidence intervals (CIs) of key indicators related to household net ownership (using a single observation per household), usage of nets the previous night, frequency of staying in the farm hut or forest, and use of nets in the farm hut or forest. Population access to an ITN was calculated as previously recommended [16]. First, the number of ITN in the household was multiplied by a factor of 2.0 to get the number of “potential ITN users”. To adjust for households with more than one net per two people, the potential ITN users was set to the de-facto population in that household. Then, the potential ITN users was divided by the number of household members as reported by the household head to determine the overall sample mean access.

### Observational studies

#### Transect walks

**Fig. 2:**
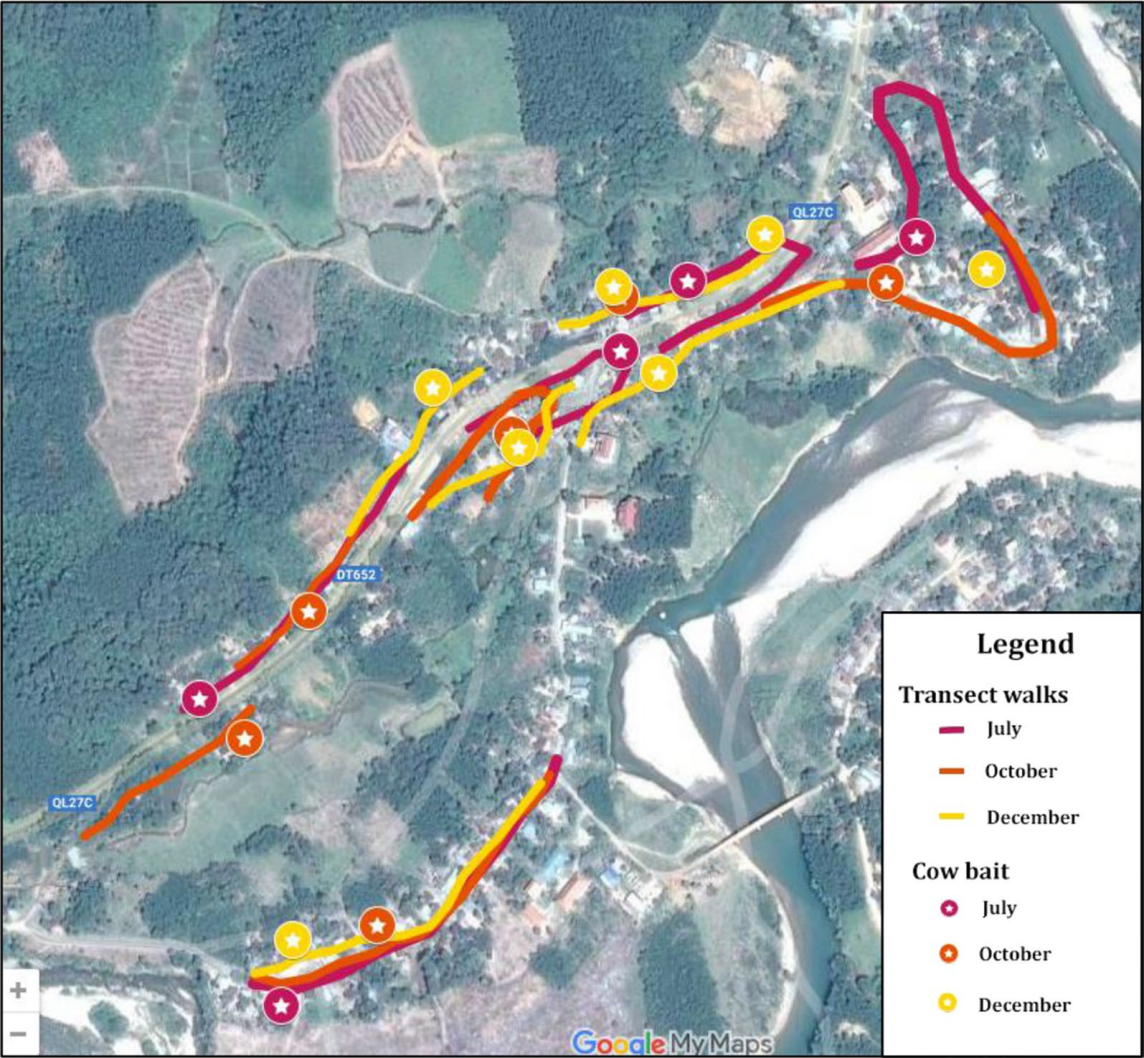
Transect walk routes walked in July, October and December collection periods. Each route corresponds to one night of observation. Routes were walked concurrent to cow-bait and human landing catch collections. Walking routes and positions of cow-bait catches varied per night in order to capture transmission risk across the commune

Concurrent to mosquito collections, transect walks were conducted during July, October and December through the study villages to observe the number of people outside their households at each hour of the night. Transect routes were purposefully selected to include major pathways through the village and past the majority of households (Fig. 2). The transects were walked on the hour, every hour from 18:00–06:00 h and the number of people observed outdoors on each hourly walk was recorded along with the type of activity being conducted. Over all observation nights, the mean number of people observed outside per hour of the evening was calculated and analysed qualitatively along with the type of activity being conducted so as to highlight reasons that took people away from the protection of nets.

#### Household net use

Concurrent to entomological collections in July, October and December, survey teams visited randomly selected households each hour of the collection evening (18:00–00:00 h) to record the number of people inside the households and the use or non-use of bednets, as well as the types of activities being conducted that kept people outside of nets. To achieve an accurate measure of number of households using nets (estimated at 85%), with 95% confidence level and 0.05 precision, the number of households to be sampled was calculated to be at least 67. In practice, the survey teams visited as many households as possible within the hourly time-frame.

Households were randomly selected from a list of households given by the local authorities using a random number generator and heads of the households were approached prior to the start of collection to obtain informed consent. Teams also observed the housing structure. Data were analysed to give estimates of the proportion of households with poor housing structure, thereby allowing entry of mosquitoes, and to give the proportion of household members using a net per hour of the evening (calculated by dividing the number of people observed under a net by the total people observed in the household per hour of observation). This provides an indication of total net use and times at which residents tended to use nets in comparison to key biting times found in the entomological collections.

### Entomological collections

#### Sampling sites

Mosquito collections were conducted during the rainy (July, October) and end of rainy season (December) when biting rates were expected to be highest, as described in Table 1. Village, farm and forest sites were collected concurrently. In the village setting, indoor HLC (IHLC), OHLC and cow-bait collections were conducted. Each night, a pair of IHLC and OHLC sites were separated by 20 m and the pair were then moved by at least 100 m the next night to attempt to capture any variation in mosquito density across the village setting. OHLC and IHLC were conducted in the farm huts and OHLC was done in the forest. Collection sites in the villages and outside were selected in July by the survey team based on previous experience of mosquito collections in the area that were known to be frequented by the villagers and where anopheline mosquitoes had previously been captured [9]. In October and December, additional collection sites in the farm huts and forest were selected from locations visited by participants in a concurrent GPS-tracking study. The methodology and results of this study will be presented elsewhere (Chavez et al., manuscript in preparation), but briefly, farm and forest-goers from Son Thai commune were purposively recruited to wear GPS trackers when they travelled into the farm hut or forest areas. Data on movement were collected for up to four days in line with the battery life of the tracker. The GPS data were then analysed to see where people had spent time during the hours 18:00–06:00 h when away from the village and these were plotted on a map. From among the sites where participants had stayed, entomological collection sites were purposively chosen based on accessibility for the survey team. Final HLC sites are shown in Fig. 1.

**Table 1:** Entomological collection nights and other activities conducted in each ecological location

Location	Month	Entomological collections			Other activities conducted
		No. of collection sites	No. of person-nights collection per month	Total no. of person-nights collection	
Village	Jul	5 (rotated per night)	5 IHLC/OHLC; 5 cow-bait	18 IHLC; 18 OHLC; 18 cow-bait	Cross-sectional survey, transect walks, household net use
	Oct	6 (rotated per night)	6 IHLC/OHLC; 6 cow-bait		
	Dec	7 (rotated per night)	7 IHLC/OHLC; 7 cow-bait		
Farm huts	Jul	1	5 IHLC/OHLC	21 IHLC (16 for extended hours); 21 OHLC (16 for extended hours)	Household net use
	Oct	3 (concurrent collection)	9 IHLC/OHLC^a^ (3 per site)		
	Dec	1	7 IHLC/OHLC^a^		
Forest	Jul	1	5 OHLC	15 OHLC (10 for extended hours)	
	Oct	1	3 OHLC^a,b^		
	Dec	1	7 OHLC^a^		
Total		18 village sites (in close proximity); 3 unique farm hut sites (one site sampled repeatedly); 3 unique forest sites		39 IHLC; 54 OHLC; 18 cow-bait (111 total)	

^a^Collection nights include extended hours of 16:00–18:00

^b^Few collection days due to heavy rain making forest sites inaccessible

*Abbreviations*: OHLC, outdoor human landing catch; IHLC, indoor human landing catch

#### Collection method

For each HLC site in the village(s), farm huts and forest two-person teams collected mosquitoes from 18:00 to 00:00 h (person 1) and 00:00 to 06:00 h (person 2). Each hour included 45 min of collection and 15 min break to prevent fatigue. One person sat with their lower legs exposed and, using an aspirator tube and torch, collected any mosquitoes landing on their legs and transferred them into glass tubes labelled by date, location and hour of collection [17]. The cow-bait catch was conducted using a single cow in a tent-trap in Bo Lang village. Mosquitoes resting on the inside of the tent were collected using aspirators every hour from 18:00 to 06:00 h. Collection positions were changed each collection night to attempt to capture transmission risk across the village setting (Fig. 1). At the end of each collection period, mosquitoes were transported to the National Institute of Malariology, Parasitology and Entomology (NIMPE) Laboratory, Ha Noi, Viet Nam, for processing.

Following the initial collection period in July 2016 (see Table 1), collection times were extended to 16:00–06:00 h in the farm hut and forest locations due to the high volume of mosquitoes caught in the early evening period. Biting rates were calculated per month and in total across the three months. Since the extended hours of 16:00–18:00 h were conducted for fewer nights, the total biting rate across all three months was calculated by adding (i) the total number of mosquitoes captured during 18:00–06:00 h divided by the total number person-nights; and (ii) the total number of mosquitoes captured during 16:00–18:00 h divided by the number of person-nights with the extended collection hours. For example, there were 21 OHLC collection nights in total in the farm huts and 16 collection nights with the extended hours, if 100 anopheles were captured in total including 18 in the period 16:00–18:00 h, the biting rate overall would be (82/21) + (18/16). This makes the appropriate adjustment for the reduced number of collection nights in this extended period.

### Laboratory analysis

Specimens were processed in the laboratory according to time, study site and method of collection. All mosquitoes were morphologically identified by experienced entomologists.

Heads and thoraxes of all collected mosquitoes were then analysed by nested-polymerase chain reaction (PCR) to detect *Plasmodium* infection. DNA extraction was conducted using a QIAamp DNA micro kit (Qiagen, Germantown, Maryland, USA). Amplification of *Plasmodium* DNA was performed using the primers PL1473F18 and PL1679R18 to target the *18S* rRNA. Separate identification of each of the four human-infecting *Plasmodium* species, *P. falciparum*, *P. vivax*, *P. ovale* and *P. malariae* was conducted using a set of primers as described by Snounou et al. [18]. To identify any simian *Plasmodium* species, samples were also tested with a primer set as described in Lee et al. [19]. Amplification products were subsequently cloned using the Original TA cloning kit (Invitrogen, Carlsbad, CA, USA) according to the manufacturer’s instructions and sequenced (GenoScreen, Lille, France).

### Entomological data analysis

Analysis was conducted for each site and method of collection separately to include abundance of *Anopheles*, nightly and hourly biting rates, anopheline infection status and entomological inoculation rate (EIR). Rate of exophagy was calculated as HBR_O_/(HBR_O_ + HBR_I_), where HBR_O_ and HBR_I_ are the human outdoor and indoor biting rates, respectively. Rate of zoophagy was calculated as, CBR/(CBR+ HBR_O_) where CBR is the cattle biting rate and HBR_o_ the outdoor human biting rate.

### Meteorological data collection

Per entomological collection night, temperature and relative humidity were recorded in the village location using a HOBO® weather data logger (Onset, Cape Cod, Massachusetts, USA). Linear regression analysis was conducted comparing number of anopheles captured per night per catch method against mean nightly temperature and relative humidity. Monthly rainfall data were retrospectively collected from the meteorological station in Khanh Vinh District and compared to monthly biting rates.

## Results

### Cross-sectional survey

#### Sample demographics

A total of 308 households were included in the survey with data gathered on 548 individuals (1–2 persons per household). The majority of people were from Bo Lang village (as opposed to Giang Bien) and a higher proportion were female (59.1%) compared to male (40.9%, Additional file 1: Table S1). The majority of individuals were from the Coho ethnic group, had not received any education and worked as farmers.

#### Village net and IRS coverage

Ownership of nets and ITNs was very high. Almost all households owned at least one ITN (99.0%; 95% CI: 97.2–99.8%) and over three-quarters (76.3%; 95% CI: 71.1–80.9%) of households owned sufficient ITNs (one ITN per two people in the household, Table 2). Only 9 households reported receiving IRS in the previous 12 months but in combination with sufficient ITN/LLIN ownership this meant that 77.6% (95% CI: 72.5–82.1%) of households were protected by vector control measures. Population access to an ITN/LLIN the previous night was very high at 91.5% and reported usage of bednets the previous night among the two selected individuals per household was also high at 95.6% (95% CI: 93.5–97.1%); data were not gathered on the type of net used.

**Table 2:** Village coverage of nets and IRS

Survey indicators	%	95% CI
Household (HH) indicators (*n* = 308)		
HHs with at least one net	99.7	98.2–100
HHs with at least one ITN^a^	99.0	97.2–99.8
HHs with at least one LLIN^a^	96.8	94.1–98.4
HHs with at least one net (any) net per 2 people	79.9	75.0–84.2
HHs with at least one ITN^a^ per 2 people	76.3	71.1–80.9
HHs with at least one LLIN^a^ net per 2 people	49.0	43.3–54.8
IRS in previous 12 m	2.9	1.3–5.5
HHs with sufficient ITNs^a^ and/or IRS in previous 12 months	77.6	72.5–82.1
Population access to ITN^a^ in HH	91.5	89.4–93.5
Person indicator (*n* = 548):		
Use of net (any net) previous night among surveyed individuals	95.6	93.5–97.1

^a^ITN (or pyrethroid-only nets) covers both conventionally treated nets that rely on periodic re-treatment of nets by dipping into an insecticide formulation, and factory-treated LLINs made of netting material with insecticide incorporated within or bound around the fibres. LLINs are defined as retaining their effective biological activity for at least 20 WHO standard washes under laboratory conditions and three years of recommended use under field conditions [20]

#### Farm and forest-going habits and net use

The vast majority (92.5%; 95% CI: 90.0–94.6%) of individuals owned (or their family owned) a forest farm plot (Table 3). These farm plots were located a median walking time of two hours away from the village home (median = 120 min, interquartile range (IQR) 60–180 min), although some could walk up to 6 h (range 10–360 min). There were 71.9% (95% CI: 67.9–75.6%) of participants who sometimes slept in the farm huts overnight while 33.2% (95% CI: 29.3–37.3%) would sometimes stay overnight in the forest.

**Table 3:** Net use among individuals that sleep overnight at the farm or in the forest

Survey indicators	%	95% CI
Individual or their family owns a farm field (*n* = 548)	92.5	90.0–94.6
Sleep overnight in farm field (*n* = 548)	71.9	67.9–75.6
Bring net to the field (*n* = 394)	70.8	66.1–75.3
Use net in field (*n* = 394)		
No	30.7	26.2–35.5
Regularly	44.4	39.4–49.5
Sometimes	24.9	20.7–29.4
Go to forest and stay overnight (*n* = 548)	33.2	29.3–37.3
Bring net to forest (*n* = 182)	48.4	40.9–55.9
Use net in forest (*n* = 182)		
No	86.8	81.0–91.4
Regularly	12.1	7.7–17.7
Sometimes	1.1	0.1–3.9

Beyond the village, net ownership and use was much lower. Among the 394 individuals interviewed that sometimes slept overnight in the farm huts, 44.4% regularly used a bednet and 24.9% sometimes used a bednet (Table 3). Only 12.1% of forest-goers regularly used a net overnight in the forest and 1.1% sometimes used a net. Data on type of net used were not gathered.

There were no significant differences in the proportion of individuals that sometimes slept in the farm huts or used nets in the farm huts by sex, age group, ethnic group or education level (data not shown). There were no significant differences by sex, age or ethnic group in terms of who went overnight to the forest or used a net in the forest except that people with secondary education or higher were less likely to go to the forest than individuals with a lower level of education (data not shown).

### Observational studies

#### Transect walks

In Son Thai commune, 417 people were observed outside over the 16 nights of transect walks (mean per night = 26.06). We estimate each transect walk covered < 10% of the village, thus covering a maximum population of 200 (10% of 2015 total population size), thus leading to around one-tenth of people observed outside during night-time hours. In the evenings people were mostly observed outdoors until around 21:00 h working, walking or conversing with neighbours. Some people were observed outdoors later than this (until 22:00 h) while drinking alcohol with friends. In the morning people were observed up and outdoors from 5 am cooking, eating breakfast and working (Table 4). In the farm huts no one was reported to be observed outside their huts during these hours on the collection nights.

**Table 4:** Frequency and activities of people observed outdoors (indicated with ‘+’) during each hourly transect walk in the Son Thai commune

Start time of transect walk	Mean no. of people per night	Activities conducted								
		Bathing	Listening to radio	Conversing	Alcohol drinking	Eating	Cooking	Working	Walking	Other
18:00	13.56	**+**	**+**	**+**		**+**	**+**	**+**	**+**	**+**
19:00	5.50	**+**		**+**		**+**		**+**	**+**	**+**
20:00	4.00			**+**				**+**	**+**	**+**
21:00	1.56			**+**				**+**	**+**	**+**
22:00	0.31				**+**					
23:00	0.00									
00:00	0.00									
1:00	0.00									
2:00	0.00									
3:00	0.00									
4:00	0.00									
5:00	1.13					**+**	**+**	**+**		
Total	26.06									

#### Household net use

**Fig. 3:**
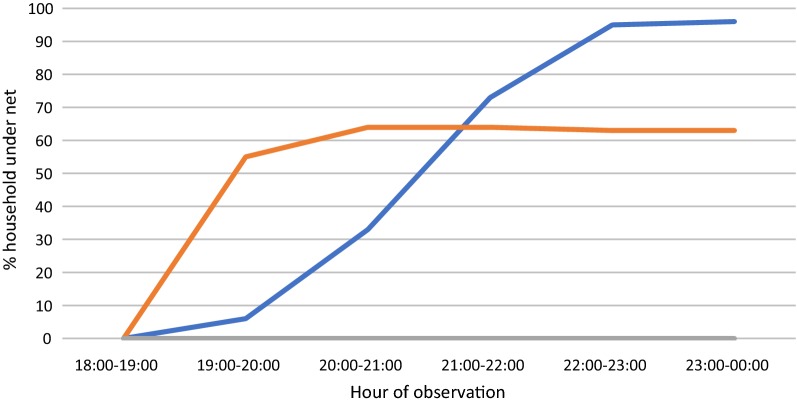
Household net use by hour of the night. Blue line: village households (mean across 110 households); orange line: farm huts (mean across 9 farm huts)

Net use was observed in village households and farm huts. In the villages, 110 households in total were observed over the three collection periods (June, *n* = 35; October, *n* = 35; and December, *n* = 40). Since monthly totals did not meet the required sample size of 67, the analysis was conducted overall and not analysed by collection month. Households were mostly made of brick (79%) and with closed walls in 96% of total households. On average, net use among people inside the households was only 6% during the 19:00–20:00 h period, rising to 33% during 20:00–21:00 h and 73% before 22:00 h (Fig. 3). From 22:00 h until midnight (the end of the observation period), net use was not universal but reached over 90%. No one was observed to be staying in the farm huts during the July collection period; however, there were six farm huts with people staying in October and three farm huts during December. In December, no one from these farm huts was observed using a net at any time, while about two thirds of people were using a net by 20:00 h in October. On average, about half of the people sleeping at the farm huts were observed using a net from about 20:00 h onwards (Fig. 3). All farm huts were made of wood or bamboo and had a partially open-walled structure.

### Entomological results

#### Abundance

**Fig. 4:**
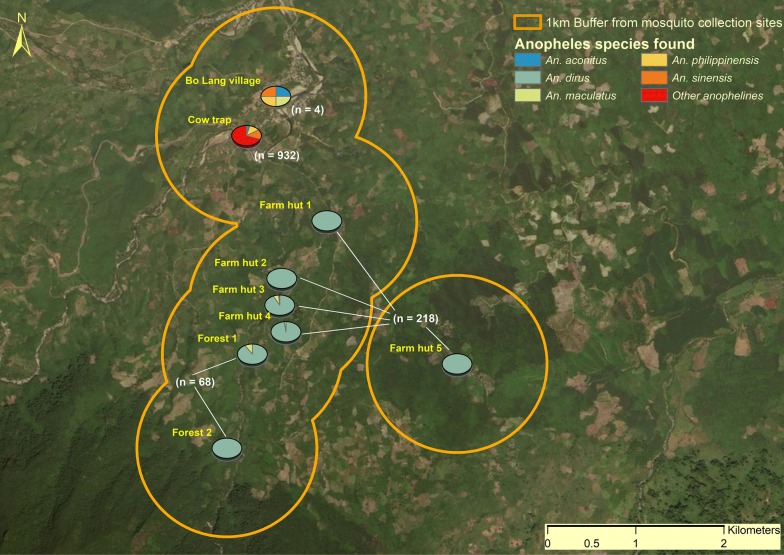
Anopheline species diversity and abundance per catch site and catch method

From a total of 111 collection nights of HLC and cow-baited collections in 3 ecological settings (Table 1) a total of 1222 anophelines were captured, including 290 (23.7%) by HLC and 932 (76.3%) by cow-bait catch (Table 5). Of the 290 anophelines captured by HLC, 93.4% were the primary vector species *An. dirus* (*s.l.*) (*n* = 271) and 4.8% were the secondary vector, *An. maculatus* (*s.l.*) (*n* = 14). Despite greater species diversity in the village site (11 species in total, including 4 by HLC), no primary vector species were captured in the village during any of the collection periods, and only a single secondary vector, *An. maculatus* (*s.l.*), was captured by OHLC (0.06 bpn; Fig. 4 and Table 5). The 932 anophelines captured by cow-bait included an additional 10 *An. maculatus* (*s.l.*) (0.56 bites per cow per night), giving a zoophagic ratio of 0.9, as well as nine other different species, including *An. barbirostris* (*n* = 33, 1.83 bites per cow per night).

**Table 5:** Biting rates of primary [*An. dirus* (*s.l.*)], secondary [*An. maculatus* (*s.l.*)] and other anophelines captured by HLC and cow-bait catch across three ecological sites and in each collection month

Location, catch method and anopheline species	Biting rate (*n*) of each primary and secondary vector species per collection site			
	July	October	December	Total
Village				
Cow-bait				
*An. maculatus* (*s.l.*)	1.0 (5)	0	0.71 (5)	0.56 (10)
Other anophelines	48.0 (240)	73.5 (441)	34.4 (241)	51.2 (922)
OHLC				
*An. maculatus* (*s.l.*)	0	0	0.1 (1)	0.1 (1)
Other anophelines	0.4 (2)	0.2 (1)	0	0.2 (3)
Farm hut				
IHLC				
*An. dirus* (*s.l.*)	8.8 (44)	1.7 (15)^a^	4.3 (30)	4.3 (89)
*An. maculatus* (*s.l.*)	0	0	0.3 (2)	0.1 (2)
OHLC				
*An. dirus* (*s.l.*)	9.0 (45)	0.2 (2)^a^	10.0 (70)	5.7 (117)
*An. maculatus* (*s.l.*)	0.4 (2)	0	0.9 (6)	0.5 (8)
Other anophelines	0	0	0.3 (2)	0.1 (2)
Forest				
OHLC				
*An. dirus* (*s.l.*)	5.2 (26)	0	5.6 (39)	4.5 (65)
*An. maculatus* (*s.l.*)	0.6 (3)	0	0	0.2 (3)

^a^Heavy rain was experienced in October (see section on rainfall data)

In the farm huts, 218 anophelines were captured (from three different species), of which 94.5% were *An. dirus* (*s.l.*) (*n* = 206), with indoor and outdoor biting rates of 4.25 and 5.65 bpn, respectively (exophagic ratio = 0.57), and 4.6% (*n* = 10) were *An. maculatus* (*s.l.*) with indoor and outdoor biting rates of 0.13 and 0.47 bpn, respectively (exophagic ratio = 0.78, Fig. 4). In the forest, 68 anophelines were captured from two species, namely 65 *An. dirus* (*s.l.*) (95.6%) and three *An. maculatus* (*s.l.*) (4.4%) with outdoor biting rates of 4.53 and 0.20 bpn, respectively (Table 5).

Biting rates were much lower in October compared to both July and December in both the farm huts and forest locations (Table 5). The exophagic ratio also appeared different in each month in the farm huts so that in July, indoor and outdoor biting were comparable, in October outdoor biting was much lower, and in December outdoor biting was more than two-times as high as indoor biting.

#### Hourly biting rates of primary vector species

**Fig. 5:**
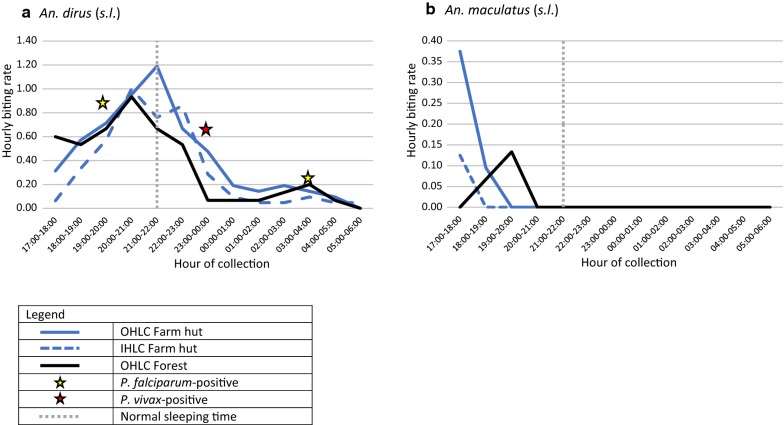
Hourly biting profile of *An. dirus* (*s.l.*) (**a**) and *An. maculatus* (*s.l.*) (**b**) in farm huts and forest. Plots show biting times in relation to normal sleeping time for Ra-glai ethnic group as found by Van Bortel et al. [4] in 2010 and at what time positive *Plasmodium* specimens were captured

Almost all biting by *An. dirus* (*s.l.*) (80.6% in the farm huts and 86.2% in the forest) was found before 23:00 h (Fig. 5). Outdoor biting in the forest and indoor biting at the farm hut were highest during 20:00–21:00 h (*n* = 14 and *n* = 21, respectively). Outdoor biting in the farm hut was highest slightly later at 21:00–22:00 h (*n* = 25). For *An. maculatus* (*s.l.*), all biting was conducted before 20:00 h and was highest in the farm huts during the extended hours of collection at 17:00–18:00 h (*n* = 6 in outdoor site and *n* = 2 in indoor site). In the forest, *An. maculatus* (*s.l.*) were only caught between 18:00 and 20:00 h.

It has previously been reported that normal sleeping time in farm huts of a similar, predominantly Ra-glai, community was 21:00 h. Here, 48% of biting by *An. dirus* (*s.l.*) and *An. maculatus* (*s.l.*) was conducted before 21:00 h in the farm huts (45% of *An. dirus* (*s.l.*) and 100% of *An. maculatus* (*s.l.*) biting).

#### Infectivity

There were 555 anopheline mosquitoes tested for malaria infection by PCR, including all 271 *An. dirus* (*s.l.*). Three *An. dirus* (*s.l.*) were found to be sporozoite positive (1.1%), two with *P. falciparum* and one with *P. vivax*. All were obtained in July collections. The positive *P. falciparum-*infected specimens were caught outdoors at the farm hut during 03:00–04:00 h and outdoors in the forest at 19:00–20:00 (indicated by yellow stars in Fig. 5). The positive *P. vivax* specimen was caught in the outdoor farm hut during 23:00–00:00 h (indicated by a white star in Fig. 5). This resulted in an EIR for *P. falciparum* in the outdoor farm hut site of 17.8 infectious bites per person per year and of 25.3 infectious bites per person per year in the forest specifically from *An. dirus* (*s.l.*). The EIR for *P. vivax* from *An. dirus* (*s.l.*) in the forest site was also 25.3 infectious bites per person per year.

### Temperature and rainfall

**Fig. 6:**
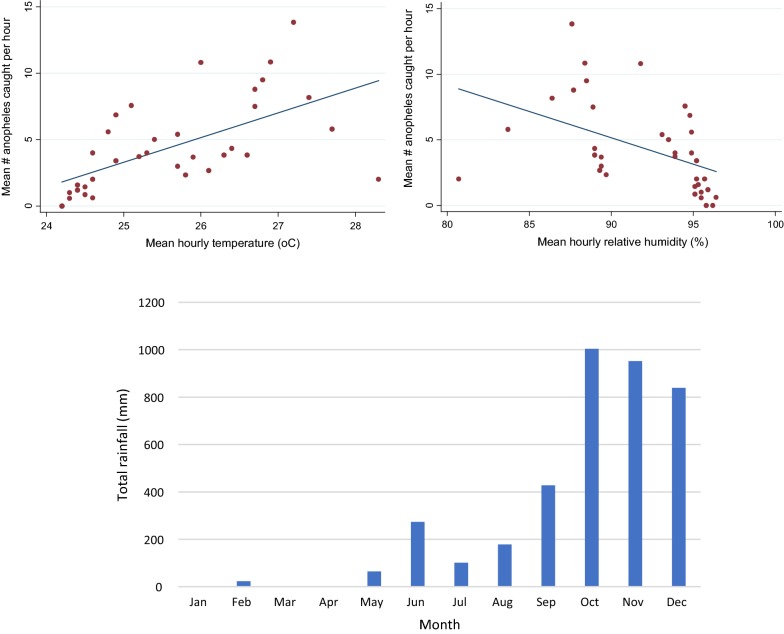
Meteorological factors affecting anopheline abundance. Top: association of abundance of *Anopheles* caught by cow-bait catch with temperature (left) and humidity (right). Bottom: total monthly rainfall during 2016 in mm

Data were collected on temperature and relative humidity during each mosquito catch period. Linear regression analysis found associations between number of anopheles caught by cow-bait with temperature and relative humidity (Fig. 6). As temperature increased, the number of *Anopheles* caught also increased (regression coefficient 1.86, *P* < 0.001). Conversely, as relative humidity increased, the number of anopheles caught went down (correlation coefficient − 0.40, *P* = 0.004). No association was found for human landing catch in farm huts or forest.

Monthly rainfall data show heavier rainfall in October when anopheline numbers were lower and which also reduced the number of collection nights able to be completed by the survey team in farm and forest locations (Fig. 6).

## Discussion

This study provides evidence on potential factors contributing to sustained transmission in this community of south-central Viet Nam. Transmission risk was minimal in the village setting where no primary vectors were detected and only a few secondary vectors, almost all of which were caught by cow-bait catch as opposed to HLC. Instead, primary and secondary vectors were abundant in farm hut plots and in the forest. Improved housing and use of ITNs have previously been shown to be highly protective except where people regularly sleep in the forest [21]. The main vectors, *An. dirus* and *An. minimus*, are relatively sensitive to insecticides, although insecticide resistance has occurred in low or transmission free areas [22]. Wide use of permethrin-impregnated mosquito nets has likely had an impact in reducing the populations and survival of the endophilic and endophagic *An. minimus* A, one of the two main malaria mosquitoes in this area of Viet Nam [23].

The majority of the community, regardless of sex, age or other demographic grouping, regularly stays overnight on farm hut plots and/or in the forest where the risk of primary and secondary anopheline biting was considerably higher since *An*. *dirus* (*s.l.*) and *An. maculatus* (*s.l.*) were abundant. Biting rates in these sites are comparable to those found previously in neighbouring sites [9, 24]. We found EIR for *P. falciparum* to be highest in the farm huts, while a *P. vivax-* positive anopheline was found in the forest. Our EIRs were 8- and 10-times higher than those found in a similar setting in Ninh Thuan Province in 2004–2006, and 17- to 25-times higher than that found in a village in central Viet Nam in 1998 [4, 25]. This could be an indication of either (i) the extent of heterogeneity between sites; (ii) the variability in sampling and thus difficulty in determining transmission parameters; (iii) the fact that by moving far away from the village we have identified the areas of highest risk; or (iv) a combination of all the above. It also reflects the difficulty of malaria control in these more remote locations.

**Fig. 7:**
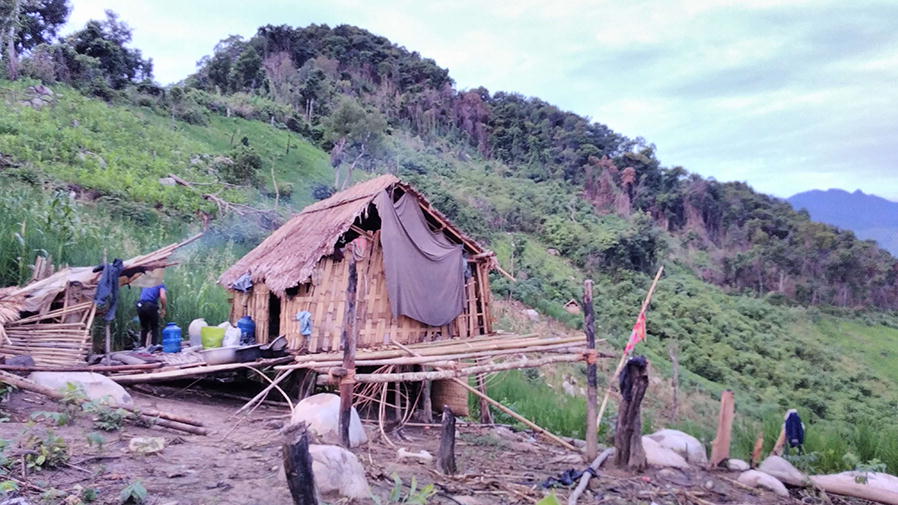
Example of local farm hut with plenty of space for entry of mosquitoes

Biting rates in the farm huts were comparable to those seen in the forest and being inside farm huts offered little to no protection since the farm hut structures were poor and freely allowed entry of mosquitoes through the walls and floors (Fig. 7). Improving housing structure or house screening has been suggested as a main intervention in areas such as this where the indoor/outdoor biting distinction is blurred [14, 26, 27]. In our study here, the prominence of indoor biting was particularly evident during a period of heavy rain in October when anophelines were driven inside and only caught by IHLC. Seyoum et al. [27] found that despite evidence of exophagy by mosquitoes, since people are generally indoors throughout the evening and night-time hours the majority of biting exposure still occurs indoors, particularly for non-users of LLINs. This has also been demonstrated in several other countries [10] although in others the greater exposure can come from outdoors [28]. In the present study, few people were observed to be staying in the farm huts during the collection periods but those that remained inside farm huts throughout the evening period where, although no infectious anophelines were observed, biting rates were high.

Despite the high mosquito abundance, less than half of people reported regularly used a net in the farm huts. This is much lower than net use rates found in a previous study in central Viet Nam where 45% of the predominantly Ra-glai population stayed overnight at the farm and over 90% of these used a bednet [8]. Since people mostly remain inside farm huts during the evening time, improving bednet compliance could positively impact transmission risk in these farm hut sites despite vectors showing exophagic behaviour. Gathering qualitative data from farm and forest goers as part of this study would have provided better insight on reasons for the lack of use of bednets in these sites and is a limitation of the study in its current form. One reason for lack of bednet use in the farms may be that ownership of sufficient ITNs in the village was sub-optimal, thereby not leaving spare nets to carry to the farms. The bednet policy in Viet Nam is to annually treat nets owned by households, replenish damaged nets and distribute LLINs so that households have maximum of two people per net. However, this only accounts for sleeping in the village house. To cover areas where there is the highest transmission risk, nets need to be distributed to cover the additional sleeping places in farm huts.

Even if net use in farm huts could be realised, early biting activity could hamper their effect since 45% of biting by *An. dirus* (*s.l.*) and 100% of biting from *An. maculatus* (*s.l.*) was observed before 21:00 which has previously been reported as the normal sleeping time for people of a similar community at their farm huts [4]. This is very similar to findings in this same study in which 45% of the *Anopheles* bites were acquired before sleeping time in the forest, and 64% before sleeping time in the village [4]. There is some evidence to suggest (although inconclusive) that shifts occur amongst the main malaria vectors to outdoor biting and earlier biting following scale-up of ITNs which would pose a significant challenge to malaria elimination [4, 29]. Although people may reside inside farm huts during these earlier hours, they could not reasonably be expected to be under a net since household activities such as cooking need to be conducted. Furthermore, achieving net use in forest locations is not feasible since people go to the forest to hunt and forage at night-time and often just rest out in the open, and people often engage in social activities outside of the protection of nets [8]. Long-lasting insecticidal hammock nets (LLIHNs) are often discussed as a solution for controlling forest transmission; however, in this study only 48.4% of forest goers carried a net with them last time they stayed in the forest and 12.1% used one on a regular basis. Other recent research also showed that insecticide-treated hammock nets were rarely taken to the forest [8] and forest workers often cite LLIHNs as being too cumbersome to use due to poor design, damaging easily, not allowing for communal sleeping arrangements, or not suiting forest resting places and the many forest workers who work through the night [30, 31]. These human behaviours plus a propensity for key vectors to bite outdoors, could limit the effect of any scale-up of ITNs. For example, a longitudinal study in Khanh Phu commune, also in Khanh Vinh District, found that ITNs produced a 5-fold reduction in malaria in the commune as a whole, but in one hamlet which had the highest proportion of people going to sleep overnight in the forests, there was no significant change from baseline, and while *An. minimus* populations were reduced, *An. dirus* persisted in forested sites [23]. A recent study in another village (Lang Nhot) in Kanh Hoa Province questioned the efficacy of insecticide-treated nets or hammocks against *An. dirus*, as more than 50% of the bites of *An. dirus A* occurred before 22:00 h [24]. Many studies before the upscaling of bednets found that nights at farm huts and in the forest were a risk factor for malaria; however, there have been few recent studies that have looked into risks after expanded malaria control activities have been achieved [8, 20, 26, 32].

Further research is needed on the user acceptability of LLIHNs and their durability, particularly for forest workers and other mobile populations in order to assess their relevance for malaria prevention in these communities [31]. Protecting people where use of nets is not appropriate, feasible or affordable will require novel personal protection technologies that are easy to carry, require little behavioural change and that allow people to continue with their normal daily tasks. Supplementary vector control tools such as spatial repellents for use at farms and in the forest or permethrin-treated clothing for evening gatherings in hamlets and all-night forest work may have a role, although neither tool shows consistent efficacy up to now [33, 34].

An alternative could be to exploit the apparent low dispersal or resting site preference of *An. dirus* (*s.l.*) for the application of insecticide, growth inhibitor or removal trapping [23].

In contrast to previous studies conducted in Khanh Hoa, we did not capture any *An. minimus* mosquitoes, which were considered a primary vector in this region and associated with anthropophilic behaviour [13]. Apart from behavioural heterogeneity [35, 36], it is not surprising to see good control of this species since it has shown high sensitivity to this type of control. In nearby Khanh Phu commune, the *An. minimus* population virtually disappeared after the introduction of permethrin-treated bedets [23] and remained absent for the next 18 years (Ron Marchand, personal commumnication). In Assam, north-eastern India, *An. minimus* mosquitoes were not seen resting inside human dwellings after an initial three years of continuous LLINs distribution [37]. The LLIN-based intervention not only deterred entry of *An. minimus* species, but also served as personal guard against infective mosquito bites corroborated by data on human mosquito landing catches and declining trends of malaria transmission [38]. The use of public health insecticides in Nepal eliminated *An. minimus* (*s.l.*) [39] and significantly reduced populations in the Thailand peninsula and central plains, although they did remain abundant in hilly forested areas [35, 40].

There are several limitations to the present study. First, farm hut and forest locations were selected largely according to accessibility and thus were not all repeatedly sampled and did not encompass the full range of sites frequented by the community. Farm hut plots are widespread and up to 11 km or further away according to a concurrent GPS tracking study (Chavez et al., manuscript in preparation). Transmission in these sites presents a big hindrance to elimination not only because vector control measures are harder to implement and monitor, but also because febrile and sick people have less or no access to prompt health services, thereby increasing their infectiousness to vectors. Conducting multiple repeat collections at the same farm hut sites and investigating other sites further away would allow understanding of the spatial heterogeneity between farm hut sites and farm hut-to-village or forest-to-village transmission. This could be better operationalised by training the local population to conduct the mosquito catches at their own farm huts, thereby increasing the number of sites reached. It has been shown to be feasible to do this in a similar concurrent study conducted in Thailand [41] and in Zambia, decentralized community-based mosquito trapping schemes was found to be far more affordable, epidemiologically relevant and cost-effective than centrally supervised trapping schemes and may well be applicable to enhance intervention trials and even enable routine programmatic monitoring of vector population dynamics on unprecedented national scales [42].

Secondly, we are unable to conclude on the relevance of seasonality or climatic factors in this area since we did not collect over the dry season. We did attempt to explore the impact of meteorological factors on anopheles biting and found some association between biting on cattle with temperature and humidity; however, biting rates on humans were too low to detect any association. The driest month of collection was in July and both *An. dirus* (*s.l.*) and *An. maculatus* (*s.l.*) were caught in July as well as October, suggesting they could be present throughout the year and contributing to perennial malaria transmission. In the nearby Khanh Phu forest, *An. dirus* was present throughout the year and most abundant between the middle and end of the dry season, i.e. February–April ([29]; Ron Marchand, personal communication.). A previous study in a neighbouring village of Khanh Hoa Province, found that temperature variation between winter and summer was not as important in this area as it was in more northern regions and thus had lower effect on endophilic behaviour [13].

Thirdly, although the observational studies provide qualitative additional information on human behaviour it is difficult to draw quantitative conclusions since (i) the transect walks could not cover the whole village and thus the population denominator to understand how many people are outside per hour is unclear; and (ii) very few people were observed to be staying in the farm huts despite high reported use of them by the community. Both of these limitations, and the results in general, would have benefited from additional qualitative methods (e.g. interviews and focus group discussions) to further understand human evening behaviours, sleep times, seasonality of farming and reasons for or against net use.

## Conclusions

Malaria control in Viet Nam has seen great success but the ecological and human behavioural factors that contribute to diverse pockets of remaining transmission will make it difficult to eliminate transmission. The biting rates, EIRs and human behavioural patterns are similar to previous studies and demonstrate the difficulty in addressing these aspects in a timely manner where populations are remote. These factors will need to be addressed with new personal protection tools that will require little behaviour change and thus be highly accessible and feasible for use by the population. Until more research is conducted on the effectiveness of many of these tools, the gaps beyond the village control setting will likely remain.

## Supplementary information

**Additional file 1: Table S1.** Demographics of people included in the cross-sectional survey.

## Data Availability

The data that support the findings of this study are available from the Malaria Consortium but restrictions apply to the availability of these data which were used under license for the present study, and so are not publicly available. Data are, however, available from the authors upon reasonable request and with the permission of the Malaria Consortium.

## Abbreviations

bpn: bites per person per night
CI: confidence interval
EIR: entomological inoculation rate
GMS: Greater Mekong Subregion
HLC: human landing catch
IEC: information, education, communication
IHLC: indoor human landing catch
IQR: inter-quartile range
IRS: indoor residual spraying
ITN: insecticide-treated net
LLIN: long-lasting insecticidal net
NIMPE: National Institute of Malariology, Parasitology and Entomology
NMCP: National Malaria Control Programme
OHLC: outdoor human landing catch
RMT: residual malaria transmission
