# Supplementary material for: Characterising residual malaria transmission in forested areas with low coverage of core vector control in central Viet Nam
Source: Parasit Vectors. 2019 Sep 18;12:454. doi: 10.1186/s13071-019-3695-1 (PMC6751671; doi:10.1186/s13071-019-3695-1)
Supplement: Supplementary file 1 — Additional file 1: Table S1. Demographics of people included in the cross-sectional survey. [file 13071_2019_3695_MOESM1_ESM.docx]

Additional file 1: Table S1. Demographics of people included in the cross-sectional survey

| Survey indicators | n | % | 95% CI |
| --- | --- | --- | --- |
| Village |  |  |  |
| Bo Lang | 333 | 60.77 | 56.60-64.78 |
| Giang Bien | 215 | 39.23 | 35.22-43.40 |
| Age group (years) |  |  |  |
| <5 | 28 | 5.11 | 3.55-7.31 |
| 5-17 | 160 | 29.2 | 25.53-33.16 |
| 18+ | 360 | 65.69 | 61.60-69.56 |
| Sex |  |  |  |
| Male | 224 | 40.88 | 36.82-45.06 |
| female | 324 | 59.12 | 54.94-63.18 |
| Ethnic group |  |  |  |
| Coho (Trin) | 455 | 83.03 | 79.64-85.95 |
| Raglei | 77 | 14.05 | 11.38-17.23 |
| Kinh | 13 | 2.37 | 1.38-4.05 |
| Other | 3 | 0.55 | 0.18-1.69 |
| Education |  |  |  |
| None | 245 | 44.71 | 40.58-48.91 |
| Elementary | 131 | 23.91 | 20.51-27.67 |
| Primary | 150 | 27.37 | 23.79-31.27 |
| Secondary and above | 22 | 4.01 | 2.65-6.03 |
| Occupation |  |  |  |
| Farmer | 364 | 66.42 | 62.35-70.27 |
| Collect forest products | 4 | 0.73 | 0.27-1.93 |
| Student | 89 | 16.24 | 13.38-19.58 |
| Other | 91 | 16.61 | 13.71-19.97 |
